# Follow-up conditions of care and associated factors among adult hypertensive patients during COVID-19 at West Arsi public health facilities, Southeastern Ethiopia: A multi-center cross-sectional study

**DOI:** 10.3389/fpubh.2022.1018686

**Published:** 2022-11-16

**Authors:** Ahmed Hiko, Nega Assefa, Zerihun Ataro, Addisu Sertsu, Elias Yadeta, Teganu Balcha, Abdulkerim Amano, Henock Asfaw, Deribe Bekele Dechasa, Kabtamu Nigussie, Lemesa Abdisa

**Affiliations:** ^1^School of Nursing and Midwifery, College of Health and Medical Sciences, Haramaya University, Harar, Ethiopia; ^2^Department of Medical Laboratory Sciences, College of Health and Medical Sciences, Haramaya University, Harar, Ethiopia

**Keywords:** attendance and follow-up, hypertension, COVID-19, associated factors, Ethiopia

## Abstract

**Background:**

Coronavirus disease (COVID-19) is a viral-borne infection caused by the SARS-CoV-2 virus. Aside from the morbidity and mortality effects, it leaves the majority of hypertensive patients untreated and vulnerable to uncontrolled hypertension.

**Objective:**

The purpose of this study was to assess follow-up conditions of care and its associated factors among adult hypertensive patients during COVID-19 in public health facilities of West Arsi, Southeastern Ethiopia.

**Methods:**

A health facility-based retrospective cross-sectional study was conducted among 423 adult hypertensive patients in the West Arsi public health facilities from July 5 to August 6, 2021. A systematic random sampling technique was used to recruit the study participants. A pretested structured face-to-face interviewer and medical records were used to collect sociodemographic variables, basic clinical features, and follow-up data. The follow-up conditions of care were assessed using 12 items with “yes or no” questions. Then, based on the mean value of the items, the follow-up conditions of care were dichotomized into good and poor. As a result, the follow-up condition was good if the score was greater or equal to the mean, and poor unless otherwise. To investigate parameters related with follow-up conditions of care, bivariable and multivariable logistic regression analyses were used. A 95% confidence interval and a *p*-value of 0.05 were used to indicate a significant association.

**Results:**

The rate of poor follow-up conditions of care during COVID-19 was 29% (95% confidence interval: 24.9–33.4%), according to this study. Age ≥ 60 years (AOR = 3.55; 95% CI: 2.09–6.03), transportation problem (AOR = 2.43; 95% CI: 1.28–4.61), fear of COVID-19 (AOR = 3.34; 95% CI: 1.59–7.01), co-morbidity (AOR = 1.93; 95% CI: 1.14–3.26) and physical distancing (AOR = 2.43; 95% CI: 1.44–4.12) were significantly associated with poor follow-up conditions of care.

**Conclusions:**

In our study, 29% of the participants had poor follow-up care conditions. When compared to WHO recommendations, the findings of this study may explain poor follow-up care conditions. To enhance patients' follow-up treatment, evidence-based target interventions should be designed and executed, taking into account individuals at high risks, such as those over the age of 60 and those with comorbidities, and identifying additional risk factors.

## Introduction

COVID-19 is one of the most common causes of death associated with severe acute respiratory syndrome 2 (SARS-COVID-2) (1). Since its inception, the global shutdown has caused global population challenges in isolation and quarantine measures, affecting nearly 620 million confirmed cases and nearly 6.54 million deaths as of 25 September 2022 (2).

The non-communicable diseases worsen the severity of COVID-19 and raise the risk of death (3). Hypertension is the leading cause of global public health problems (4). It is the leading risk factor for global disease burden, accounting for 7% of global disability-adjusted life years (DALY) (5). Hypertension affects 31.1% of the worldwide population, with Ethiopia accounting for 30.2% (6). Hypertension is a risk factor for cardiovascular disease and the leading cause of mortality (7). According to the World Health Organization (WHO), its complications account for 94 million deaths worldwide, with ~17 million deaths from cardiovascular disease occurring each year (7). According to one study, hypertension complications cause 45% of heart disease deaths and 51% of stroke deaths (8).

Poor follow-up care is a situation in which patients are unable to receive the WHO-recommended usual follow-up care service within the planned appointment time frame for a variety of reasons. Typical follow-up treatment includes Bp measurement, medication refilling, lifestyle change recommendations, monitoring medication adherence, reviewing laboratory work, screening cardiovascular risks, and so on. When the target blood pressure was reached, WHO recommended varied checks at 3 to 6 month intervals, depending on the patients' health status. Individuals with higher BPs require shorter intervals (every 1 or 2 months), and an emergency visit is required if BP is 180/110 mmHg (9, 10).

Bp measurements are necessary for hypertension screening, diagnosis, and therapy. With restricted physical access to patients during COVID-19, healthcare providers are unsure how to measure blood pressure and analyze cardiovascular risk factors (11). Some patients avoid or postpone seeking care due to their fear of contracting COVID-19, and the ability to treat hypertensive patients has been jeopardized (12). Because of COVID-19, every system was altered, resulting in increased morbidity and mortality (13, 14).

According to the WHO disease burden and mortality estimates, non-communicable disease deaths in Africa increased from 22.8% in 2000 to 34.2% in 2016 (15). COVID-19, on the other hand, increases the burden of mortality by 13.7% in hypertensive patients (16). Border restrictions and lockdowns disrupted 64% of health services, lost patients from follow-up, and decreased medicine supplies, especially in low and middle-income countries (LMICs) (17, 18). Even for measured blood pressure status, there were 87.6% physical examinations, 47.6% laboratory tests, and 50% assessments of end-organ damage and cardiovascular risks (12). In addition, the epidemic increases the severity of hypertension problems such as ischemic heart disease, stroke, cardiovascular disease, and mortality (18, 19).

Around the world, uncontrolled hypertension causes 500,000 fatalities each year (4). According to research done in SSA, 77.4% of people had uncontrolled blood pressure. The incidence of uncontrolled hypertension varied significantly between studies, ranging from 11 to 70% (20, 21). When compared to before the COVID-19 epidemic, the prevalence of uncontrolled hypertension has skyrocketed (22). Despite COVID-19 challenges, numerous efforts have been implemented to enhance follow-up conditions of treatment and address other consequences of hypertension. A few of these included the use of telemedicine, connecting patients with neighboring medical centers, and extending the time between drug refills to reduce potential harm from subpar follow-up conditions of treatment (23–25). Despite various research on hypertension in Ethiopia, no investigations on follow-up care conditions and associated factors have been conducted. Again, the rate of follow-up care was unknown since the hypertensive patient treatment had been discontinued, drug supply had been disrupted, and all systems had changed as a result of COVI-19. Therefore, this study aims to evaluate the follow-up conditions of care and its associated factors among adult hypertensive patients during COVID-19 in public health facilities of the West Arsi zone, Southeastern Ethiopia.

## Materials and methods

### Study design and setting

A health facility-based retrospective cross-sectional study was conducted from July 5 to August 6, 2021. From all public health facilities, Kuyera specialized referral hospital, Melka Oda general hospital, Dodola general hospital, Abbosto health center, and Awasho health center were selected by simple random sampling method. The data retrieval period was from March 13, 2020, to March 14, 2021, for secondary data. Shashemene is the capital city of the West Arsi zone, which is located 250km from Addis Ababa. The overall population of this zone was estimated to be 2,520,875 according to the 2017 Central Statistical Agency population census, of whom 50.8% were women and 13.02% of those living there were urban residents (26).

### Populations and sampling procedure

The study included all adult hypertensive patients who had been taking anti-hypertensive medications for at least 6 months and were eligible for the study to assess follow-up conditions of care. But Patients with cognitive impairment, newly diagnosed, and incomplete medical records were excluded from the study. The sample size calculation was done using a single population formula. Since there was no previous research on the subject, the following assumptions were taken into account while determining the sample size: 95% confidence interval, 5% margin of error, and 50% prevalence of poor follow-up conditions of care since there was lack of published similar study on a similar topic. The total sample size increased to 423 after adding a 10% non-response rate. The following public health institutions had a total of 1,605 patients under follow-up care: Kuyera specialized referral hospital (520), Melka oda general hospital (352), Dodola general hospital (544), Abbosto health center (103), and Awasho health center (86). The average number of patients visiting a health institution each month was calculated by looking through the registration books of each facility's medical records, and then, 423 participants were proportionally allocated to each facility. Finally, from Kuyera referral hospital, Melka Oda general hospital, Dodola general hospital, Abbosto health center, and Awasho health center, respectively, 137, 93, 143, 27, and 23 people were hired. Following the determination of the K value, a systematic random sampling technique was used to choose research participants. The first case was chosen by lottery, and the remaining individuals were recruited and added one at a time.

### Data collection tools and methods

Due to the lack of standard tools (24, 27), the questionnaires were created by evaluating prior research in a way that incorporated all the factors that may achieve the study objectives. They were then adapted for the local context. To gather information from research participants, it was written in English and then translated into Afan Oromo, the local language. The tool includes socio-demographic data (age, sex, religion, educational level, marital status, place of residence, and monthly income), fundamental clinical data (family history, comorbidity, and time since diagnosis), and follow-up conditions of care-related questions (patients attendance, medication refilled, inpatient visit, prior consultation, the form of consultation, access to medication, etc.). To evaluate patients' attendance at the medical institution, blood pressure measurements, and medication refills, a checklist was also employed to analyze their medical records. By using Cronbach's alpha (α), the internal consistency of the questionnaire was further examined, and 12 follow-up items received a score of 0.85. The data was gathered using a structured face-to-face interviewer-administered questionnaire. Two health officers served as facilitators and three BSc nurses served as data collectors. Throughout the data-collecting period, all COVID-19 safety procedures were followed.

### Outcome measurement and operational definitions

There were two categories for the follow-up conditions of care: good and poor. Twelve “yes-no” questions were used to assess the follow-up conditions of care. From twelve items, two of them (unable to obtain prescription drugs and unable to obtain an in-person appointment) were negatively written, but before the analysis, they were recorded in the opposite direction to facilitate computation with other positive items. Following computation using SPSS, the mean cutoff follow-up conditions of care was eight with SD ± 0.1. Following that, the overall score for the follow-up conditions of care was divided into two categories: good for people who scored higher than or equal to the mean, and poor unless otherwise stated.

**Good follow-up conditions**: Participants in the study who achieve results above or equal to the mean on the follow-up conditions of care questions are in good follow-up circumstances (28, 29).

**Poor follow-up conditions:** Participants in the study who achieve results below the mean on the follow-up conditions of care questions are in poor follow-up circumstances (28, 29).

**Medical disruptions**: If an individual had to miss or cancel previously scheduled appointments or is unable to get an in-person appointment to see their provider (30).

**Prescription drug disruption**: If an individual is unable to obtain the prescription drugs they need or have to stop taking drugs exactly as prescribed to extend the supply (30).

**Uncontrolled hypertension:** If BP ≥150/90 mmHg in hypertensive patients aged 60 years or ≥140/90 mmHg for patients aged <60 years and all hypertensive patients with diabetes mellitus (DM) or chronic kidney disease (CKD) based on the average of three measurements (31).

**Older:** Those individuals with an age great or equal to 60 years.

**Younger**: Those individuals below the age of 60.

**Physical distancing:** Keeping a distance of 2 m from one another and limiting activities outside the home.

**Fear of COVID-19:** Anxiety and stress related to COVID-19 disease.

**Isolation:** Staying at home and avoiding contact with others.

### Data quality controls

To reduce prejudice, data collectors were hired from outside the data collection area. To ensure a common understanding, the principal investigator trained data collectors and supervisors for 2 days on the research's objectives, components, how to choose study participants, ethical issues, and a basic description of data gathering. Before the actual data collection, a pretest on 5% of the total sample was performed in the Negelle Arsi health center to improve data quality. Last but not least, significant input was included in the survey. The data collectors were under the supervision of supervisors. Every day, the supervisors rechecked the questionnaire's accuracy to address one issue at a time.

### Statistical analysis

Epi data version 3.1 software was used to enter the data, which was then exported and analyzed using SPSS version 26. Variables were compiled using descriptive statistics (frequency distributions, mean, median, standard deviation, and calculation of variables). A descriptive frequency analysis was performed to look for any missing values and outliers. To determine the linear correlations among independent variables, multicollinearity was examined using variance inflation factors and a tolerance test. As a result, no variables in this study had a VIF > 10 or a tolerance test of 0.1. Hosmer-Lemeshow and Omnibus tests were used to assess the model's goodness of fit. Hosmer-Lemeshow (*p* = 0.473) and Omnibus tests (*p* = 0.000) showed a good model fit. To account for all potential confounders, in binary logistic regression, variables with *p*-values ≤ 0.25 at 95% confidence intervals (CI) were taken into consideration and integrated into the multivariable regression model. An adjusted odds ratio with a 95% confidence interval was deemed statistically significant in the multivariable model at a *p*-value of ≤0.05.

## Results

### Socio-demographic characteristics of participants

The research included a total of 409 participants, yielding a 96.69% response rate. More than half of the study participants 228 (55.7%) were male. The median age (±SD) was 52 ± 14.38 years and 142 (34.7%) fall within the age above 60 years. Around 26.9% attended Elementary school, 35.9% were farmers in occupation, and 53.1% were urban dwellers.

In terms of essential clinical characteristics, 91 (22.2%) people had a family history of hypertension. One hundred eighty-one responders (44.3%) reported co-morbidities with various conditions. The median illness duration for hypertensive patients was 4.98 years, with an interquartile range of 4.5 years (Q1 = 2.5 and Q3 = 7). The disease duration varied from 1 to 20 years. Of all responders, 343 (83.9%) were using one or more anti-hypertensive medications (Table 1).

**Table 1:** Socio-demographic characteristics and basic clinical characteristics of hypertensive patients at West Arsi public health facilities, 2021 (*N* = 409).

**Variables**	**Category**	**Frequency**	**Percentage %**
Age in years	20–29	19	4.6
	30–39	61	14.9
	40–49	82	20
	50–59	105	25.7
	≥60	142	34.7
Educational status	Unable to read and write	105	25.7
	Able to read and write	90	22
	Primary education (1–8)	110	26.9
	Secondary education (9–12)	66	16.1
	Higher education	38	9.3
Occupation	Farmer	147	35.9
	Merchant	113	27.6
	Government employee	45	11
	Housewife	103	25.2
	Others	1	0.2
Residence	Urban	217	53.1
	Rural	192	46.9
Comorbidity	No	228	55.7
	Diabetes	136	33.3
	Cardiovascular disease	24	5.9
	Hyperlipidemia	4	1
	Others^**^	17	4.2
Number of anti-hypertensive drugs	1–2	343	83.9
	2–4	66	16.1
Duration of the disease since diagnosed	<3 years	101	24.7
	3–5 years	161	39.4
	>5 years	139	34

*, others for others religion believers, Wakefata, Adventist, Catholic and Traditional believers;

** , others for others disease, Stroke, Urinary tract infection, Asthma, and eye disease.

### Follow-up conditions of care

Poor follow-up conditions of care had an overall rate of 29% (95% confidence interval: 24.9–33.4%). In terms of the follow-up conditions of care, 347 (84.8%) people visited a medical institution, 84.3% had their blood pressure taken, 84.1% had their prescriptions renewed, and 83.1% received counseling. Participants in the research who had a prior consultation <3 months ago totaled 359 (75.6%). Ninety-two (22.5%) people experienced medical issues, whereas 338 (82.6%) people were able to get prescription drugs. Seventy-four (18.1%) had to cease taking prescription medications exactly as directed. Three hundred forty (83.1%) had access to medicines from medical facilities, 375 (91.7%) had access to in-person consultations with medical experts, 384 (93.9%) had appointments for in-person consultations, and 171 (41%) had appointments for telephone consultations with medical professionals (Table 2).

**Table 2:** Follow-up condition of care among adult hypertensive patients at West Arsi public health facilities during COVID-19, Ethiopia, 2021 (*N* = 409).

**Variables**	**Category**	**Frequency**	**Percentage %**
Patients attend health facilities	Yes	347	84.8
	No	62	15.2
BP measured	Yes	345	84.4
	No	64	15.6
Medication refilled	Yes	347	84.8
	No	62	15.2
Advice given	Yes	346	84.6
	No	63	15.4
Prior consultations	<3 months	309	75.6
	>3 months	100	24.4
Unable to obtain an in-person appointment	Yes	92	22.5
	No	317	77.5
Unable to obtain prescription drugs	Yes	75	18.3
	No	334	81.7
Able to get prescription medication	Yes	338	82.6
	No	71	17.4
Able to get access to medication	Yes	340	83.1
	No	69	16.9
Get access to an in-person visit	Yes	375	91.7
	No	34	8.3
Did you have an appointment with a healthcare provider in person for your high blood pressure?	Yes	384	93.9
	No	25	6.1
Did you have an appointment either by phone or computer for high blood pressure?	Yes	171	41.8
	No	238	58.2
The overall follow-up condition of care	Good	290	70.9
	Poor	119	29.1

### Reasons why prescription drugs are difficult to get

Of those who had to cease taking prescription medications exactly as directed or were unable to acquire them, 24 (32%), 72 (96%), 35 (46.7%), 51 (68%), and 42 (56%) were due to closed hospital/clinics, fear of COVID-19, closed pharmacy, fear of using public transit, and the rising cost of medications, respectively, as indicated in Table 3.

**Table 3:** Reasons why prescription drugs are difficult to get among adult hypertensive patients at West Arsi Public health facilities during COVID-19, Ethiopia, 2021 (*N* = 75).

**Variables**	**Frequency**	**Percentage %**
**Reasons why prescription drugs are difficult to get (*****N*** **= 75)**		
Because of closed hospitals/clinics	24	32
Due to fear of COVID-19	72	96
Pharmacy closed	35	46.7
Due to fear of using public transport	51	68
The Increased cost of medicine	42	56

### Reasons why some people are unable to obtain medicine

**Figure 1:**
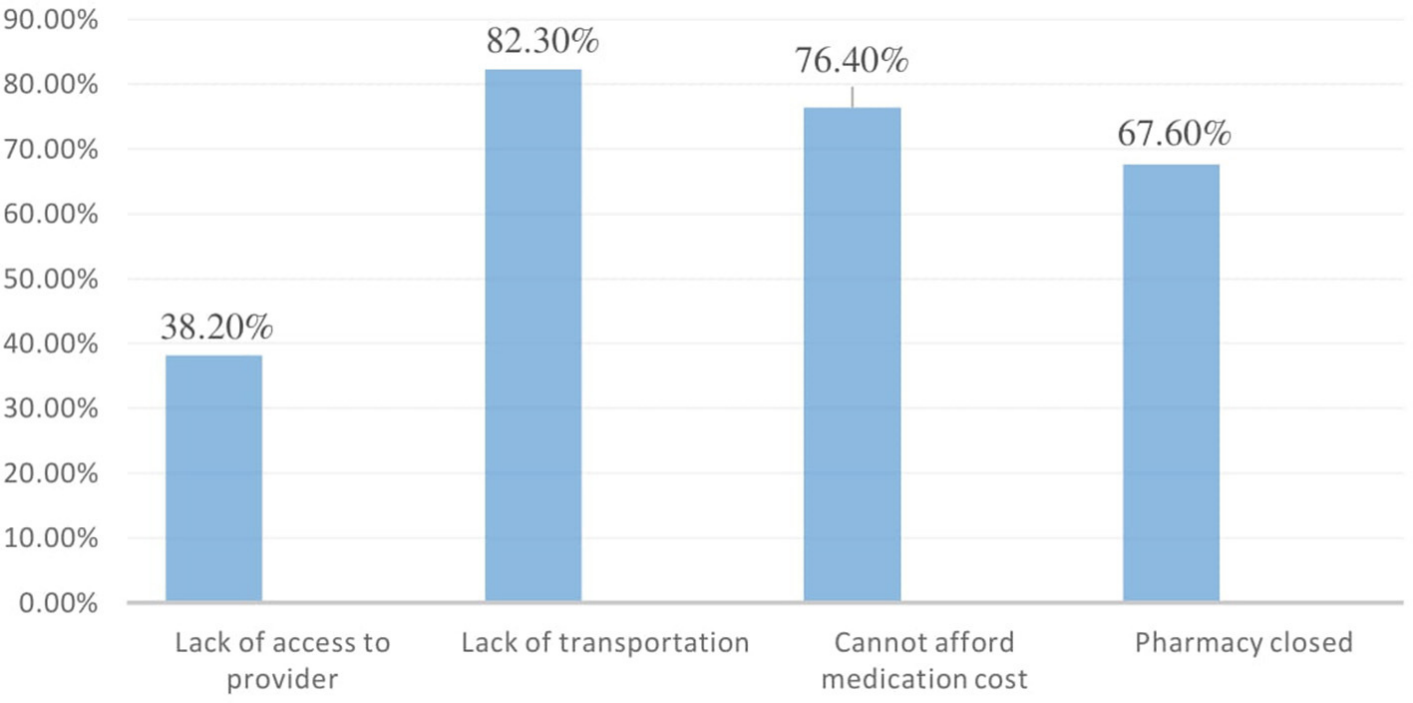
Reasons why some people cannot get medicine during COVID-19, among hypertensive patients at West Arsi public health facilities, Ethiopia, 2021.

Of all respondents, 340 (83.1%) needed to access medication from health facilities, while the remaining respondents were unable to do so for a variety of reasons, including difficulty accessing providers (38.2%), difficulty traveling (82.3%), inability to pay for medication costs (76.4%), and pharmacy closure (67.6%), as indicated in Figure 1.

### Time to refill medications

**Figure 2:**
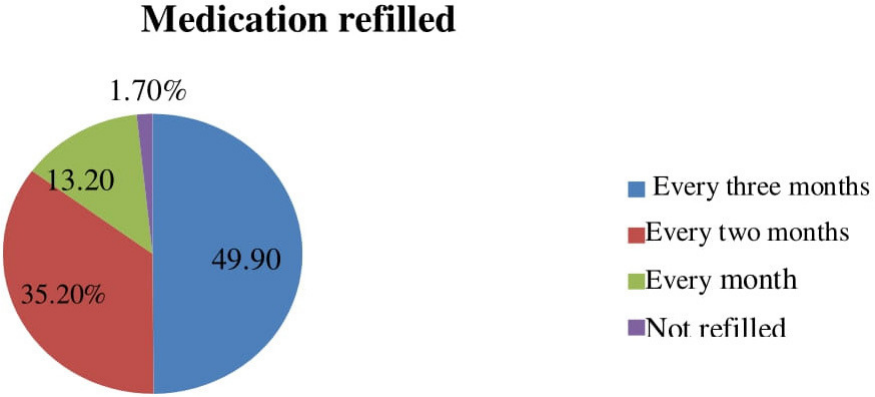
Medication refilled during COVID-19, among hypertensive patients at West Arsi public health facilities, Ethiopia, 2021.

Of all respondents, 204(49.9%) said their prescriptions were renewed every 3 months, while only 1.7% said they weren't (Figure 2).

### The potential reasons for reduced access to healthcare

**Figure 3:**
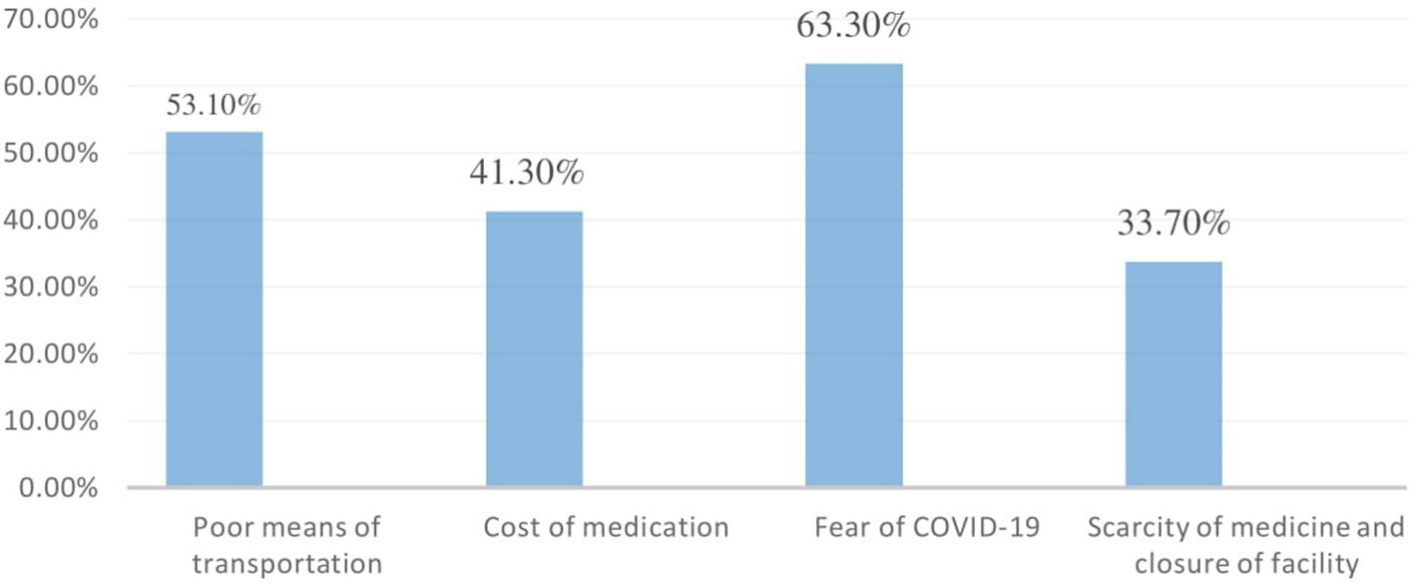
The potential cause of reduced access to healthcare among adult hypertensive patients on follow-up at West Arsi public health facilities, Ethiopia, 2021.

According to the findings of this survey, 217 (53.1%) insufficient transportation, 169 (41.3%) pharmaceutical costs, 263 (63.3%) fear of COVID-19, and 138 (33.7%) shortage of necessary medicine and closure of some health facilities are possible reasons that reduce access to health care (Figure 3). One hundred twenty-nine (31.5%) reported that their health condition declined due to a lack of access to health care.

### COVID-19 preventive measures related

According to this study, 244 (59.7%) of study participants reported lockdown measures, followed by physical distancing 188 (46%), and isolation 76 (18.6%) were factors that affected seeking health care services.

### Factors associated with poor follow-up conditions of care

In a binary logistic regression analysis, poor follow-up conditions of care were substantially correlated with age, educational status, monthly income, site of residence, comorbidities, transportation issues, fear of COVID-19, physical distance, and isolation. However, gender, religion, occupation, and marital status were not associated with the outcome variable. To control confounders, all variables with a *p*-value of <0.25 in the bivariable analysis were further considered in the multivariable logistic regression analysis.

Age, fear of COVID-19, transportation issues, comorbidities, and physical distance were all substantially linked with poor follow-up conditions of care on multivariable logistic regression analysis with a *p*-value < 0.05. Patients over the age of 60 were 3.55 times more likely than younger people to have poor follow-up conditions of care (AOR = 3.55, 95% CI: 2.09–6.03). Those who dread COVID-19 are 3.34 times more likely than their counterparts to have poor follow-up conditions of care (AOR = 3.34, 95% CI: 1.59–7.01). When compared to patients without transportation issues, patients with transportation issues had a 2.43 times higher likelihood of having poor follow-up conditions of care (AOR = 2.43, 95% CI: 1.28–4.61). Comorbidities increased the likelihood of having poor follow-up conditions of care by 1.93 times compared to counterparts (AOR = 1.93, 95% CI: 1.14–3.26). When the physical distance was present, the odds of poor follow-up conditions of care were 2.43 times greater than when it wasn't (AOR = 2.43, 95% CI: 1.44–4.12) at *p*-value 0.05 (Table 4).

**Table 4:** Bivariable and multivariable logistic regression analysis depicting factors associated with poor follow-up conditions of care among adult hypertensive patients at West Arsi public health facilities, Ethiopia, 2021 (*N* = 409).

**Variables**		**Follow-up conditions**		**COR (95% CI)**	**AOR (95% CI**
	**Poor (%)**	**Good (%)**			
Age	Older	70 (49.3)	72 (50.7)	4.3 (2.75, 6.8)^***^	3.55 (2.09, 6.03)^***^
	Young	49 (18.4)	218 (81.6)	1	1
Place of residence	Rural	73 (38)	119 (62)	2.28 (1.47, 3.53)^***^	0.91 (0.48,1.73)
	Urban	46 (21.2)	171 (78.8)	1	1
Fear of COVID-19	Yes	105 (40.5)	154 (59.5)	6.62 (3.62, 12.11)^***^	3.34 (1.59, 7.01)^**^
	No	14 (9.3)	136 (90.7)	1	1
Transportation problem	Yes	94 (42.4)	125 (57.6)	4.6 (2.76, 7.33)^***^	2.43 (1.28, 4.61)^**^
	No	27 (14.1)	165 (85.9)	1	1
Comorbidity	Yes	71 (39.2)	110 (60.8)	2.42 (1.56, 3.75)^***^	1.93 (1.14, 3.26)^*^
	No	48 (21.1)	180 (78.9)	1	1
Physical distancing	Yes	75 (39.9)	113 (60.1)	2.67 (1.72, 4.15)^***^	2.43 (1.44, 4.12)^**^
	No	44 (19.9)	177 (80.1)	1	1
Educational status	No formal education	41 (39)	64 (61)	1.86 (1.16, 2.97) ^*^	1.34 (0.72, 2.50)
	Formal education	78 (25.7)	226 (74.3)	1	1
Isolation	Yes	40 (44.4)	50 (55.6)	2.43 (1.49, 3.96)^***^	1.70 (0.94, 3.09)
	No	79 (24.8)	240 (75.2)	1	1
Monthly income	< 1,500 ETB	89 (33.7)	175 (66.3)	1.93 (1.2, 3.11)^**^	1.57 (0.78, 3.15)
	>1,500 ETB	30 (20.8)	114 (79.2)	1	1

COR, crude odds ratio; CI, confidence interval; AOR, adjusted odds ratio; significant at

* p < 0.05;

** p < 0.01;

*** p < 0.001.

## Discussion

According to this study, 29% of patients had poor follow-up conditions. This discovery was consistent with observations from Northwest Ethiopia (32). However, the results of this study were lower (70%) than those of the Addis Ababa study (23). These may be explained by variations in temporary lockdown measures, attitudes about COVID-19, and methodological variations, particularly in terms of data collection techniques and research timeframe. In the current study, patients over the age of 60 years were more likely to have poor follow-up conditions of care. This matched the findings of the Addis Ababa investigation (23). The elderly are at a higher risk of COVID-19 case fatality, no caregivers are available for senior patients, and there are comorbid conditions present, which discourage the patients from attending treatment follow-up care (11, 32). Another reason for this might be the physiological outcome of weakened immunity in older persons, which increases the severity of COVID-19 risk factors and age-related reasonable comorbidity, both of which have a substantial influence (33–37). As a result, patients become concerned about the outbreak, and medical facilities are compelled to close. However, the findings of a research conducted in Islamabad, Pakistan, contradict our findings (38). This chasm may be produced by the patient's understanding of their ailment as well as the length of their therapy. For example, elderly patients getting long-term care adhere to their treatment plan and timeline. As a result, they have favorable follow-up medical conditions.

In this study, patients who dread COVID-19 were more likely than their counterparts to have poor follow-up conditions of treatment. This was in line with a prior investigation carried out in Addis Ababa (23). The outbreak provided ample evidence that anxiety, the high-risk perception among hypertension patients, and widespread media misinformation all played significant roles (39).

Patients with transportation problems had a greater risk of receiving subpar follow-up treatment than patients without such issues. This was consistent with the Addis Ababa study (23). The conceivable defense may be that there is a government lockdown and that there is a COVID-19 contracting scare. For instance, the government announced the COVID-19 preventive procedure after the pandemic emerged to stop the virus from spreading throughout the nation. The restriction of transportation services was one of the procedures. As a result of the risk posed by COVID-19, transportation services are drastically reduced. It was said that restrictions on transportation for health reasons had existed in the past (40). On the other hand, transportation and mobility have emerged as possible hotspots for the virus' transmission, particularly in areas with dense populations and few resources (41). The need for patient follow-up care may decline as a result. Another reason could be that most people travel from far-flung rural areas to get health care.

In this research, comorbidity increased the likelihood of having subpar follow-up conditions of care. This contradicted the findings of a study conducted in Islamabad, Pakistan (38). This disparity might be attributed to the research participants' differing degrees of knowledge about the risk of getting COVID-19 and its severity. Furthermore, COVID-19 is likely to impact those with underlying medical illnesses more frequently, hasten the course of the disease, and considerably increase mortality across all age groups, particularly those over the age of 60 (42). As a result, patients may become less reluctant to seek medical treatment.

Physical distance increased the likelihood of poor follow-up conditions of care compared to no physical distance. However, there were no research that supported this discovery. This might be due to physical separation, which encourages patients to stay at home, and medical personnel limiting the number of patients admitted to the hospital in order to prevent the coronavirus from spreading.

Another reason might be related to the reduction of essential clinical services, loss of patient follow-up, fragmentation of care, the decline in direct healthcare worker participation, and restrictions on patient access to health facilities because of enforced physical distance (43).

### Implications for clinical practice

The results of our study have significant implications for practice and policy. Treatments that improve patients' follow-up care circumstances at health institutions must be designed and implemented. We discovered numerous characteristics linked with poor follow-up conditions of care in this study, including age, fear of COVID-19, transportation issues, the existence of comorbidities, and physical distance. As a result, any intervention aimed at enhancing follow-up care conditions might be beneficial. Interventions to improve follow-up conditions of care may include providing free medical care, educational interventions to educate patients about the benefits of regular treatment follow-up, providing awareness on COVID-19, family support, providing better services in healthcare settings, sending reminders to patients *via* phone calls, SMS, emails, and other means, linking patients to the nearby health facility, and so on (44–48).

To avoid the future advancement of their disease and its consequences, patients must be educated on how to carefully follow the directions offered by healthcare professionals. Because appropriate follow-up conditions of care result in optimal blood pressure control, healthcare practitioners should emphasize the necessity and purpose of attending follow-up appointments during first consultations. This would enable them to undertake required monitoring and medication optimization to provide the best potential treatment outcomes for patients (38). Because inpatient therapy was not possible due to the hazards of COVID-19 transmission, sending reminders through phone call, SMS, and email was an optional treatment approach to ensure continuity of care. Better health care was highly indicated by identifying persons at high risk, such as older patients and those with comorbidity, because this group of patients was extensively attacked by COVID-19 and the severity of the disease itself was worse in this group of patients. This results in better follow-up care. In general, during COVID-19, there is an urgent need for a systematic strategy to improve patient follow-up care, with a mix of a patient-centric approach and any digital and mobile technologies strongly suggested (49). The distribution and cause-effect relationship should be researched further in future studies.

## Strength and limitations

The study's strength is that it involved many centers and included various hypertension individuals with various features. This broadens the applicability of the findings and the representativeness of sample groups. A whole dataset of medical records and in-person interviews were employed as data acquisition strategies to improve data accuracy and completeness. Physical distance was one of the variables that was included to determine how it affected the conditions of care for subsequent visits. However, there are certain limitations to this study. The study's design was its main shortcoming. Due to the retrospective nature of the study, memory bias may result from study participants' inability to recollect their former experiences. We employ a self-reported instrument that might introduce social desirability bias. Additionally, the study excluded adult hypertension patients who were receiving follow-up care at private medical institutions.

## Conclusions

This study found that 29% of patients had poor follow-up conditions of treatment. When compared to WHO recommendations, the findings of this study may explain poor follow-up care conditions. To increase patients' follow-up care, evidence-based target interventions should be designed and implemented, taking into account individuals at high risks, such as those over the age of 60, those with comorbidity, and identifying other risk factors. And to stop the situation from getting worse, healthcare providers and other stakeholders should work in collaboration.

## Data availability statement

The raw data supporting the conclusions of this article will be made available by the authors, without undue reservation.

## Ethics statement

The studies involving human participants were reviewed and approved by Haramaya University, College of Health and Medical Sciences, an Institute of the Health Research Ethics Review Committee (IHRER). The patients/participants provided their written informed consent to participate in this study. All research participants who were 18 years old at the time of the study gave their informed, voluntary, written, and signed permission after being informed of its goals, objectives, advantages, and disadvantages. Those who were unable to read have had their consent read to them and signed with their fingerprint.

## Author contributions

AH, NA, ZA, and AS took part in the idea's inception, the proposal's development and revision, the analysis of data, and the writing up of the findings. EY, TB, AA, HA, DD, KN, and LA have participated in the analysis and manuscript write-up. All authors contributed to the article and approved the submitted version.

## Conflict of interest

The authors declare that the research was conducted in the absence of any commercial or financial relationships that could be construed as a potential conflict of interest.

## Publisher's note

All claims expressed in this article are solely those of the authors and do not necessarily represent those of their affiliated organizations, or those of the publisher, the editors and the reviewers. Any product that may be evaluated in this article, or claim that may be made by its manufacturer, is not guaranteed or endorsed by the publisher.

## Abbreviations

AOR: Adjusted Odds Ratio
BP: Blood Pressure
CI: Confidence Interval
CVD: Cardiovascular Disease
DALY: Global Disability-adjusted Life Years
HCW: Health Care Worker
LMCIs: Low and Middle-income Countries
NCD: Non-communicable Disease
SSA: Sab-Sahara Africa
WHO: World Health Organizations.
