# Supplementary material for: Follow-up conditions of care and associated factors among adult hypertensive patients during COVID-19 at West Arsi public health facilities, Southeastern Ethiopia: A multi-center cross-sectional study
Source: Front Public Health. 2022 Nov 16;10:1018686. doi: 10.3389/fpubh.2022.1018686 (PMC9708880; doi:10.3389/fpubh.2022.1018686)
Supplement: Supplementary file 1 [file Table_1.DOCX]

**English Version of Questionnaire**

Part I. Socio-demographic Characteristics of Respondents

Instruction: Please circle the number in front of the option you choose and fill in the blank space that best describes you on the right side of the table.

Q.ID­______________

| S. no | Variables | Response |
| --- | --- | --- |
| 101 | Age | ­­­­­­­­­­­­­____________ |
| 102 | Sex | 1. Male 2. Female |
| 103 | Religion | 1. Orthodox 2. Muslim 3. Protestant 4. Other(specify)_______ |
| 104 | Marital status | 1. Single 2. Married 3. Divorced 4. Widowed |
| 105 | Educational status | 1. unable to read and write 2. able to read and write 3. 1-8 grade 4. 9-12^th^ 5. Higher educations |
| 106 | Occupations | 1. Farmer 2. Merchant 3. Government employ 4. Housewife |
| 107 | Place of residence | 1. Urban 2. Rural |
| 108 | Monthly income | _________________ |

Part II. Basic clinical characteristics related Questions

| S.no | Variables | Response |
| --- | --- | --- |
| 201 | Does the patient have a family history of hypertension? | 1. Yes 2. No |
| 202 | Specific comorbidity that the patient has? | 1. No 2. DM 3. CVD 4. Hyperlipidemia 5. Others(Specify)___________ |
| 203 | What is the duration of disease since diagnosed? | _________Years Or __________Months |
| 204 | How many anti-hypertensive drugs the patient is taking? | ________________ |

Part III. Hypertensive patient’s monthly follow-up conditions of care related Questions

a. Checklist

| S.no | Variables | During the first 6 months  COVID-19 | Last 6 month  during COVID-19 |
| --- | --- | --- | --- |
| 301 | Did the patient attend the health facility during COVID-19? | 1. Yes 2. No |  |
| 302 | Was Bp measured for patients at health facilities during COVID-19? | 1. Yes 2. No |  |
| 303 | Was Medication refilled for patients during COVID-19? | 1. Yes 2. No |  |

b. Questionnaire

| S. no | Variables | | Response | | Skip |
| --- | --- | --- | --- | --- | --- |
| 304 | Does advice given to you on treatment and lifestyle modification during COVID-19? | | 1. Yes 2. No | |  |
| 305 | Does the health care provider encourage you to monitor your blood pressure at home during | | 1. Yes  2. No | |  |
|  | COVID-19? | |  | |  |
| 306 | What is your opinion about anti-hypertension medication refill during COVID-19? | | 1. Easy and not costly  2. Easy and costly  3. Not easy due to any reason  4. Did not refill for more than one month | |  |
| 307 | When did you have prior consultation? | | 1. <3months 2. 3-6months 3. 7-12months 4. No consultation | |  |
| 308 | What is the form of consultation during COVID-19? | | 1. Face to face consultation 2. Telephone consultation 3. Video call | |  |
| 309 | How do you monitor your blood pressure during the outbreak? | | 1. Home Bp monitoring 2. Other methods of Bp monitoring 3. Not monitoring | |  |
| 310 | Do you have a will to attend your health care visit during COVID- as usual, one? | | 1. Yes, 2. No | |  |
| 311 | If no to QNo.310 why do you not have the will to attend your health care? | | | | |
|  | Fear of being exposed to covid-19 | | | 1. Yes, 2. No |  |
|  | Fear of being exposed during transportation | | | 1. Yes 2. No |  |
|  | Difficulty in making appointments due to limited availability | | | 1. Yes 2. No |  |
|  | Lack of transportation | | | 1. Yes 2. No |  |
| 312 | Had you unable to obtain an in-person appointment to see your provider during COVID-19? | | | 1. Yes 2. No |  |
| 313 | Had you been unable to obtain the prescriptions drugs you need since the outbreak? | | | 1. Yes 2. No |  |
| 314 | Since the COVID-19 outbreak, are you able to get your prescription medications? | | | 1. Yes 2. No | If yes skip Q.No.315 |
| 315 | Why you are not able to get your medications? | Clinics/hospital closed | | 1. Yes 2. No |  |
|  |  | Fear of contracting coronavirus | | 1. Yes 2. No |  |
|  |  | Pharmacy closed | | 1. Yes 2. No |  |
|  |  | Afraid to use public transportation | | 1. Yes 2. No |  |
|  |  | Increased price /cost | | 1. Yes 2. No |  |
| 316 | Have you got access to medication from the health facility during COVID-19? | | | 1. Yes 2. No | If yes skip Q.No.317 |
| 317 | If no to QNo.316 why do you stop taking your medications? | | | | |
|  | Lack of access to the provider to obtain medication refill | | 1. Yes 2. No | |  |
|  | Lack of transportation | | 1. Yes 2. No | |  |
|  | I can’t afford medication cost | | 1. Yes 2. No | |  |
|  | Pharmacy is closed | | 1. Yes 2. No | |  |
| 318 | Had you got access to an in-person visit during COVID-19? | | 1. Yes 2. No | |  |
| 319 | During the COVID-19 outbreak, did you have an appointment with a health care provider in person for your high blood pressure? | | 1. Yes 2. No | |  |
| 320 | During the COVID-19 outbreak, did you have an appointment either by phone or computer for high blood pressure? | | 1. Yes 2. No | | If no skip Q.No.321 |
| 321 | How did you meet your provider? | | 1. By phone 2. By video 3. Both phone and video | |  |
| 322 | For how many months medication is refilled for you during the Outbreak? | | ____________Months | |  |
| 323 | From the following on which your health care professional encourage you to do at your home since the outbreak? | | | | |
|  | Encourage health eating | | 1. Yes 2. No | |  |
|  | Encourage medication compliance | | 1. Yes 2. No | |  |
|  | Encourage calling care provider with questions and concerns | | 1. Yes 2. No | |  |
|  | Ensure access to medications | | 1. Yes 2. No | |  |
| 324 | What is the reason that makes decreases access to health care during COVID-19? | | | | |
|  | Poor means of transportation | | 1. Yes 2. No | |  |
|  | Cost of medicine | | 1. Yes 2. No | |  |
|  | Fear of exposure to coronavirus during hospital visits | | 1. Yes 2. No | |  |
|  | Refusal of a health care worker to attend patients for fear of contracting virus | | 1. Yes 2. No | |  |
|  | Scarcity of required medicine and closure of some health care facilities | | 1. Yes 2. No | |  |
| 325 | What do you feel about your health conditions during COVID-19? | | 1. No change 2. Deteriorated 3. Improved | |  |
| 326 | Which of the following does not allow you to attend your follow-up clinics during Outbreak? | | | | |
|  | Lockdown measures | | 1. Yes 2. No | |  |
|  | Physical distancing | | 1. Yes 2. No | |  |
|  | Isolation | | 1. Yes 2. No | |  |

Thank you for your participation!!!!!
